# Prof. Horatio R. Storer’s Lctures

**Published:** 1867-04

**Authors:** 


### Prof. Horatio R. Storer's Lctures.

It will be observed by notice in advertisement sheet, that Dr. H. R. Storer will give lectures upon the *Surgical Diseases of Women*, at his rooms in Hotel Pelham, Boston, in June next. Dr. Storer was with Dr. Simpson of Edinburgh, as his assistant in practice, during 1854–55, and selected by him, as one of the editors of his Obstetric works, and has bestowed much time and study upon the diseases of women. With ample opportunities for observation, both at home and in other countries, there can be no doubt but his lectures will embody all the recent advances made in this important department of practice. Prof. Storer is well and widely known both as a distinguished writer and teacher, and we have no doubt that many members of the profession who desire perfect knowledge in this department will gladly avail themselves of the opportunity which is now afforded them of obtaining it.

